# Antibiotic Susceptibility of Aerobic and Facultative Anaerobic Gram-Negative Rods in Hong Kong and Implications on Usefulness of Ceftazidime-Avibactam and Ceftolozane-Tazobactam

**DOI:** 10.3390/antibiotics13090802

**Published:** 2024-08-24

**Authors:** Jade L. L. Teng, Elaine Chan, Tsz Tuen Li, Tsz Ying Kwan, Ka Fai Chan, Wing Ho Li, Viki W. K. Tang, Man Lung Yeung, Susanna K. P. Lau, Patrick C. Y. Woo

**Affiliations:** 1 Faculty of Dentistry, The University of Hong Kong, Hong Kong Special Administrative Region, China; llteng@hku.hk (J.L.L.T.); whli1212@connect.hku.hk (W.H.L.); 2 Department of Microbiology, School of Clinical Medicine, Li Ka Shing Faculty of Medicine, The University of Hong Kong, Hong Kong Special Administrative Region, Chinapmlyeung@hku.hk (M.L.Y.); 3 State Key Laboratory of Emerging Infectious Diseases, Li Ka Shing Faculty of Medicine, The University of Hong Kong, Hong Kong Special Administrative Region, China; 4 Department of Clinical Microbiology and Infection Control, The University of Hong Kong-Shenzhen Hospital, Shenzhen 518053, China; 5 Carol Yu Centre for Infection, Li Ka Shing Faculty of Medicine, The University of Hong Kong, Hong Kong Special Administrative Region, China; 6 Centre for Virology, Vaccinology and Therapeutics, Hong Kong Science and Technology Park, Hong Kong Special Administrative Region, China; 7 Doctoral Program in Translational Medicine and Department of Life Sciences, National Chung Hsing University, Taichung 402, Taiwan; 8 The iEGG and Animal Biotechnology Research Center, National Chung Hsing University, Taichung 402, Taiwan

**Keywords:** antibiotic susceptibility, ceftazidime-avibactam, ceftolozane-tazobactam, multidrug resistance, ESBL-producing Enterobacterales

## Abstract

Due to the increasing resistance of aerobic and facultative anaerobic Gram-negative rods, ceftazidime-avibactam and ceftolozane-tazobactam have been launched in the market in the last few years. In this study, we analyzed the susceptibility pattern of the major aerobic and facultative anaerobic Gram-negative rods in Hong Kong for ceftazidime-avibactam, ceftolozane-tazobactam, four other broad-spectrum antibiotics commonly used in Hong Kong and colistin. For 300 isolates collected from January to December 2021, non-ESBL-producing Enterobacterales, ESBL-producing Enterobacterales and *Pseudomonas aeruginosa* were highly susceptible to ceftazidime-avibactam (all 100%) and ceftolozane-tazobactam (98.7%, 99.7% and 94.3%). For 32 archived ESBL-producing *Klebsiella pneumoniae* isolates collected between January 2014 and March 2023, all were susceptible to ceftazidime-avibactam and ceftolozane-tazobactam. For 101 archived carbapenemase-producing Enterobacterales, their susceptibilities to ceftazidime-avibactam and ceftolozane-tazobactam varied depending on the type of carbapenemase produced. Both had high activities against OXA-producing strains (97.1% and 76.5%, respectively) but were 100% resistant for NDM-producing and NDM+OXA-producing strains. All KPC-producing strains were susceptible to ceftazidime-avibactam but resistant to ceftolozane-tazobactam. Ceftazidime-avibactam and ceftolozane-tazobactam are good alternatives for the management of infections caused by ESBL-producing Enterobacterales and selective strains of carbapenemase-producing Enterobacterales in Hong Kong.

## 1. Introduction

Aerobic and facultative anaerobic Gram-negative rods are major causes of a wide variety of bacterial infections. For example, they are important pathogens for hospital-acquired pneumonia and bacteremia, which has resulted in high fatalities. Traditionally, β-lactam antibiotics have formed the backbone in the treatment of most infections caused by these Gram-negative rods [1]. However, there has been a globally increasing resistance to the first and second line β-lactam antibiotics, such as ampicillin, first- and second-generation cephalosporins, amoxicillin-clavulanate and ampicillin-sulbactam [2]. As a result of the rising resistance, many of the infections caused by these Gram-negative rods have to be treated by highly potent broad-spectrum antibiotics, also known as the “big guns”, such as piperacillin-tazobactam, the third- and fourth-generation cephalosporins and the carbapenems. Obviously, the widely use of these “big guns” has resulted in the development of resistance against them in these Gram-negative rods, such as extended-spectrum β-lactamase (ESBL)-producing Enterobacterales, carbapenemase-producing Enterobacterales (CPE) and carbapenem-resistant *Pseudomonas aeruginosa*. Infections caused by these multidrug resistance organisms have led to the reuse of highly toxic antibiotics (e.g., colistin) that have rarely been used for decades [3]. In addition, they have also stimulated the development of newer antibiotics by pharmaceutical companies. In the last few years, two cephalosporin-β-lactamase combination antibiotics, namely, ceftazidime-avibactam and ceftolozane-tazobactam, have been developed and launched in the market [4,5,6,7,8,9,10]. Ceftazidime/avibactam combines a well-known cephalosporin, ceftazidime, with a new non-β-lactam β-lactamase inhibitor, avibactam; while ceftolozane/tazobactam couples a new cephalosporin, ceftolozane, with a well-established β-lactam β-lactamase inhibitor, tazobactam. Both avibactam and tazobactam target the active site of serine β-lactamases. Ceftazidime-avibactam and ceftolozane-tazobactam are both active against ESBL-producing Enterobacterales, but they have selective activities against CPE [11,12,13,14]. In particular, they have no activity against metallo-β-lactamases producers. As antibiotic resistance of bacteria changes with time, in vitro surveillance of antibiotic susceptibility patterns is of paramount importance.

Hong Kong has been an international financial center for a few decades. It has also been a hub for travelers, particularly acting as a gateway for transportation in and out of China. The recent political turmoil in Hong Kong has resulted in migration of hundreds of thousands of Hongkongers to the western world, particularly the United Kingdom. Furthermore, there is also an anticipation of increase movement of other Hong Kong residents to the Greater Bay Area located in the Guangdong Province of China for work and/or residence. These movement of people has also resulted in a movement of their bacterial flora, and hence, to antibiotic resistance genes [15,16]. Due to the importance of aerobic and facultative anaerobic Gram-negative rods in causing various infections, it is of paramount importance to understand their susceptibility patterns to the highly potent broad-spectrum antibiotics in Hong Kong in a timely manner. However, no data on the susceptibility of these bacteria to ceftazidime-avibactam and ceftolozane-tazobactam as compared to the other “big guns” are available in Hong Kong. In this study, we analyzed the susceptibility pattern of the major aerobic and facultative anaerobic Gram-negative rods in Hong Kong for ceftazidime-avibactam, ceftolozane-tazobactam, four other “big guns” commonly used in Hong Kong and colistin. The importance of the antibiotic susceptibility pattern for the choice of antibiotics for the treatment of infections in people residing in and those from Hong Kong is also discussed.

## 2. Results

### 2.1. Collection of Bacterial Strains

For the 300 non-duplicated isolates of aerobic and facultative anaerobic Gram-negative rods initially collected, their antimicrobial susceptibilities against ceftazidime-avibactam, ceftolozane-tazobactam, piperacillin-tazobactam, ceftazidime, meropenem, cefepime and colistin were evaluated by broth microdilution method. These isolates included 137 *E. coli*, 52 *K. pneumoniae*, 35 *P. aeruginosa*, nine *Enterobacter* spp., 14 *Proteus mirabilis*, two *Proteus* spp., 12 *Citrobacter* spp., 13 *A. baumannii*, 14 *Stenotrophomonas maltophilia*, seven *Morganella morganii*, one *Providencia stuartii*, two *Serratia* spp. and two *Aeromonas* spp. They were isolated from urine, respiratory samples (e.g., endotracheal aspirate, tracheal aspirate, gastric aspirate and sputum) and blood and miscellaneous samples (e.g., superficial and deep wound swab, sterile body fluids, abscesses and tissue specimen) (Supplementary Table S1). For these 300 isolates, 55 strains were confirmed as ESBL-producers, comprising 44 *E. coli*, six *K. pneumoniae*, four *P. mirabilis* and one *Proteus* spp. The sites from which these 55 ESBL-producers were isolated from are listed in Supplementary Table S1. For the additional 32 ESBL-producing *K. pneumoniae* strains (all isolated from urine) and 101 CPE strains, they were evaluated for antimicrobial susceptibilities to ceftazidime-avibactam and ceftolozane-tazobactam. The CPE isolates included 50 *E. coli*, 38 *Klebsiella* spp., six *Citrobacter* spp. and seven *Enterobacter* spp. The sites from which these 101 CPE strains were isolated are listed in Supplementary Table S2.

### 2.2. Antimicrobial Susceptibilities of Non-Multidrug Resistant (MDR) Isolates

The in vitro performance of ceftazidime-avibactam, ceftolozane-tazobactam, piperacillin-tazobactam, ceftazidime, meropenem, cefepime and colistin against the non-MDR isolates is summarized in Table 1. The results of antimicrobial susceptibilities against ceftazidime-avibactam and ceftolozane-tazobactam in Table 1 represent MICs assessed by MIC test strips. A comparison of susceptibility against ceftazidime-avibactam assessed by both MIC test strips and broth microdilution is summarized in Supplementary Table S3, which shows comparable results were achieved when assessing using either method.

Among the non-MDR *E. coli* isolates, susceptibility was highest to ceftazidime-avibactam, ceftolozane-tazobactam and meropenem (100%, 98.9% and 98.9%, respectively), with one isolate being resistant to ceftolozane-tazobactam but susceptible to both ceftazidime-avibactam and meropenem. Among the non-MDR *K. pneumoniae*, susceptibility was highest to ceftazidime-avibactam, meropenem, ceftolozane-tazobactam and cefepime (100%, 100%, 97.8% and 97.8%, respectively). Among the non-MDR *P. aeruginosa* isolates, susceptibility was highest to ceftazidime-avibactam, ceftolozane-tazobactam and colistin (100%, 94.2% and 88.6%, respectively), with two isolates resistant to ceftolozane-tazobactam but susceptible to both ceftazidime-avibactam and colistin. Among the non-MDR *Proteus* spp., susceptibility was highest to ceftazidime-avibactam, ceftolozane-tazobactam and cefepime (100%, 90.9% and 90.9%, respectively), with one isolate resistant to both ceftolozane-tazobactam and cefepime but susceptible to ceftazidime-avibactam. The susceptibility of *A. baumannii* and *Aeromonas* spp. isolates was found to be highest for ceftazidime-avibactam (100% for both), while a lower susceptibility was observed for ceftolozane-tazobactam (61.5% for *A. baumannii* and 50% for *Aeromonas* spp.). Among the non-MDR *Enterobacter* spp., *Citrobacter* spp., *M. morganii*, *P. stuartii* and *Serratia* spp. isolates, a high susceptibility was observed to ceftazidime-avibactam, ceftolozane-tazobactam and meropenem (all 100%). On the other hand, *S. maltophilia* isolates displayed high resistance to all tested antimicrobials and showed low susceptibility to ceftazidime-avibactam (28.6%) and ceftolozane-tazobactam (14.3%), while demonstrating no susceptibility to meropenem and cefepime (0% and 0%, respectively). However, it should be noted that for species groups with fewer than 10 isolates, additional data are needed to draw a more definitive conclusion.

### 2.3. Antimicrobial Susceptibilities of MDR Isolates

The antimicrobial activity of ceftazidime-avibactam, ceftolozane-tazobactam, piperacillin-tazobactam, ceftazidime, meropenem, cefepime and colistin against the 55 ESBL-producers from the study collection isolates is summarized in Table 1. Among the ESBL-producing isolates, a high susceptibility was retained towards ceftazidime-avibactam, ceftolozane-tazobactam and meropenem (100%, 97.7% and 100%, respectively, for *E. coli*; 100%, 100% and 100%, respectively, for *K. pneumoniae*; and 100%, 100% and 100%, respectively, for *Proteus* spp.). On the other hand, the cephalosporins ceftazidime and cefepime lost their antimicrobial activity on the majority of the ESBL-producing strains, with susceptibility of 36.4% and 15.9%, respectively, for *E. coli*; and 33.3% and 33.3%, respectively, for *K. pneumoniae*. The one carbapenemase-producing *E. coli* identified in the initial 300 isolates (Table 1) was resistant to all the tested antimicrobial agents, including ceftazidime-avibactam and ceftolozane-tazobactam. This isolate was positive for the *bla*_NDM_ carbapenemase gene.

For the additional 32 ESBL-producing *K. pneumoniae* isolates, they were all susceptible to both ceftazidime-avibactam and ceftolozane-tazobactam (Table 2).

As for the 101 CPE, the efficacy of the two drugs varied among the different bacterial species. The highest susceptibility to ceftazidime-avibactam and ceftolozane-tazobactam was observed for carbapenemase-producing *Klebsiella* spp. (84.2% and 42.1%, respectively), while a lower susceptibility was observed for carbapenemase-producing *E. coli* (12% and 10%, respectively), *Citrobacter* spp. (50% and 50%, respectively) and *Enterobacter* spp. (28.6% and 28.6%, respectively). Susceptibility depending on the presence of different carbapenemase genes was also observed (Table 3). Both ceftazidime-avibactam and ceftolozane-tazobactam were effective against nearly all of the OXA-producing strains (97.1% and 76.5%, respectively), while both drugs had low activities against the NDM-producing and NDM+OXA-producing strains (100% resistant). On the other hand, the KPC-producing strains were susceptible to ceftazidime-avibactam (100% susceptible) but resistant to ceftolozane-tazobactam (100% resistant).

## 3. Discussion

In this study, we show that ceftazidime-avibactam and ceftolozane-tazobactam are good alternatives for the management of infections caused by the ESBL-producing Enterobacterales in Hong Kong. The widespread use of third-generation cephalosporins around three decades ago had resulted in the emergence of ESBL-producing *K. pneumoniae* [18]. Since then, the incidence of infections caused by ESBL-producing Enterobacterales, most importantly, ESBL-producing *E. coli* and ESBL-producing *K. pneumoniae*, has dramatically increased [19]. For example, in a study in 2017, it was observed that 52.8% of the *E. coli* isolated from stool samples in healthy people residing in Hong Kong were ESBL-positive [20]. Therefore, in the local antibiotic usage guidelines in Hong Kong, the antibiotic of choice for community-acquired upper urinary tract infections is a carbapenem [21]. The increasing use of carbapenems in the treatment of infections caused by ESBL-producing Enterobacterales has inevitably resulted in the emergence of carbapenem-resistant organisms, such as CPE and carbapenem-resistant *P. aeruginosa* [22]. In this study, it was shown that most strains of Enterobacterales, including those that produced ESBL, were susceptible to ceftazidime-avibactam and ceftolozane-tazobactam (Table 1 and Table 2). This indicated that these two recently developed cephalosporin-β-lactamase combination antibiotics could be used as alternatives for the specific treatment of infections caused by ESBL-producing Enterobacterales, in order to reduce the use of carbapenems and selective pressure for the generation of carbapenem-resistant bacteria such as CPE and carbapenem-resistant *P. aeruginosa*. Nevertheless, the emergence of resistant phenotypes to these new beta-lactam/beta-lactamase inhibitor combinations would warrant implementation of antimicrobial susceptibility testing for these antibiotics, which is best carried out through rapid diagnostic methods [23,24,25].

Ceftolozane-tazobactam and ceftazidime-avibactam can be used for the treatment of infections caused by selective strains of CPE. In the last 30 years, carbapenems have usually been considered as the ultimate β-lactam group antibiotic of choice for the treatment of infections caused by multiple-resistant Enterobacterales. The emergence of CPE and its increasing incidence in causing infections globally, particularly in countries with suboptimal antibiotic auditing programs, has caused a nightmare for infectious disease specialists, intensive care physicians, etc. When a strain of Enterobacterales becomes resistant to the carbapenems, it is often also resistant to other groups of antibiotics commonly used for the treatment of Enterobacterales infections, such as the fluoroquinolones and aminoglycosides. In many cases, colistin is used for the management of infections due to these strains of CPE [26]. In this study, most of the strains of *E coli* and *Klebsiella* spp. collected were shown to be susceptible to colistin (Table 1). However, colistin is highly toxic and some groups of bacteria, such as *Proteus* spp., *Providencia* spp. and *Morganella* spp., are intrinsically resistance to colistin (Table 1) [26]. Moreover, colistin resistance, mediated by the plasmid encoded gene *mcr-1*, has been identified worldwide [26]. In the present study, it was shown that some of the strains of CPE were susceptible to ceftazidime-avibactam and/or ceftolozane-tazobactam, depending on the specific carbapenemases produced (Table 3). Specifically, both ceftazidime-avibactam and ceftolozane-tazobactam were active against most OXA producers but resistant to all NDM+OXA producers, while the KPC producers were susceptible to ceftazidime-avibactam but resistant to ceftolozane-tazobactam, indicating that ceftazidime-avibactam could be more useful than ceftolozane-tazobactam for this group of CPE. These results concurred with those of other studies [14,27,28,29,30]. For example, two studies conducted in China and the United Arab Emirates (UAE) found that 80 to 85% of carbapenemase-producing *Klebsiella* spp. were susceptible to ceftazidime-avibactam, compared to only 1.9 to 13% for ceftolozane-tazobactam. Both antibiotics had low efficacy against carbapenemase-producing *E. coli,* with 28.6% susceptible to ceftazidime-avibactam and 7.1% to ceftolozane-tazobactam. In fact, such a susceptibility pattern was in line with the underlying resistance mechanism of the corresponding genes. Similar to our findings, these studies showed that ceftazidime-avibactam possessed better activity against KPC producers, and very few NDM isolates were susceptible to ceftolozane-tazobactam and ceftazidime-avibactam. Additionally, the majority of OXA producers were susceptible to both antibiotics [27,30]. This is because OXA-48-like carbapenemases have low hydrolytic activity against cephalosporins and, therefore, the activity of ceftolozane is preserved [31,32]. All these indicated that ceftazidime-avibactam and ceftolozane-tazobactam could be useful in the treatment of selective cases of CPE infections, irrespective of their colistin susceptibility. Yet, since a significant proportion of the strains in the current cohort were NDM-positive, antibiotic susceptibility testing must be determined before using ceftazidime-avibactam or ceftolozane-tazobactam for the CPE infections. In addition, it was also shown most of the strains of *Proteus* spp., *Providencia* spp. and *Morganella* spp. that were intrinsically resistant to colistin were also susceptible to ceftazidime-avibactam and ceftolozane-tazobactam (Table 1), indicating that these two antibiotics can also be an alternative for treatment of infections caused by these organisms.

## 4. Materials and Methods

### 4.1. Bacterial Strains

Three hundred non-duplicated isolates of aerobic and facultative anaerobic Gram-negative rods were collected over a 12-month-period (January to December 2021) from Queen Mary Hospital in Hong Kong for determination of antimicrobial susceptibility against seven antibiotics. Additionally, 32 ESBL-producing isolates and 101 CPE collected between January 2014 and March 2023 from Queen Mary Hospital were retrieved for further analysis on their susceptibilities against ceftazidime-avibactam and ceftolozane-tazobactam. The isolates were derived from five different sites of patients clinically, including urine, respiratory, blood, stool and miscellaneous samples (Supplementary Tables S1 and S2). Species identification of all isolates was confirmed by matrix-assisted laser desorption ionization-time of flight (MALDI-TOF) mass spectrometry (Bruker Daltonik, Bremen, Germany). Detection of ESBL and carbapenemase production was performed as described below. Isolates tested positive for ESBL and CPE production were defined as MDR strains. All isolates were stored at −80 °C until use. Ethics approval for this study was provided by the Institutional Review Board of The University of Hong Kong/Hospital Authority.

### 4.2. Detection of ESBL Production

The inhibition zone enhancement test was performed by applying antibiotic disks of cefotaxime (CTX-30 µg) and ceftazidime (CAZ-30 µg) alone and in combination with clavulanic acid (CTX-CLA 30/10 µg or CAZ-CLA 30/10 µg) to a 0.5 McFarland standard suspension of overnight culture on Mueller-Hinton II agar (Becton Dickinson, St. Louis, MO, USA), following standard phenotypic methods performed by the clinical microbiology laboratory [33]. The plates were then incubated at 37 °C for 18 h, and the diameter of inhibition zone around each disk was measured according to CLSI guidelines. A positive ESBL phenotype was confirmed when a ≥5 mm enhancement of the zone of inhibition was observed in CTX-CLA or CAZ-CLA compared with the diameter of the zone of inhibition of the respective antimicrobial disk alone. *K. pneumoniae* ATCC 700603 and *E. coli* ATCC 25922 were used as positive and negative controls, respectively.

### 4.3. Detection of Carbapenemase Production

Similar to the method used for detecting ESBL production, the disk diffusion method was used to determine the susceptibility of the isolates to meropenem (Thermo Fisher Scientific, Waltham, MA, USA). The results were interpreted according to CLSI guidelines [17]. For isolates that displayed resistance to meropenem, they were subjected to the CARBA5-LFA (NB Biotech, Guipry, France) to detect and differentiate the five most prevalent carbapenemases families (NDM, IMP, VIM, OXA-48 and KPC) as per the manufacturer’s instructions.

### 4.4. Determination of Antimicrobial Susceptibility by Broth Microdilution Method

Antimicrobial susceptibilities of the 300 non-duplicated isolates of aerobic and facultative anaerobic Gram-negative rods against ceftazidime-avibactam, piperacillin-tazobactam, ceftazidime, meropenem, cefepime and colistin were determined by the broth microdilution method according to the CLSI guidelines [17,33]. All antimicrobial agents were purchased from Sigma-Aldrich (St. Louis, MO, USA). Stock solutions of each antimicrobial agent were prepared according to the manufacturer’s instructions. Prepared stock solutions were aliquoted and stored at −80 °C until use. Antibiotic stock solutions were diluted at a series of two-fold dilutions using cation-adjusted Mueller Hinton broth (CAMHB) and dispensed into conical-bottomed wells of tissue culture-treated 96-well microdilution plates (Eppendorf, Leipzig, Germany). Prepared microdilution plates were sealed and stored at −80 °C until use [34,35]. Isolate strains were grown on Mueller Hinton agar at 37 °C for 18–24 h and a standardized inoculum was prepared using direct colony suspension by making a saline suspension with a turbidity equivalent to 0.5 McFarland standard. The inoculum suspension was then diluted in CAMHB (to yield approximately 5 × 10^5^ CFU/mL per well after inoculation) and added to each well of the prepared antimicrobial agent-containing microdilution plate. Microdilution plates were incubated at 35 ± 2 °C for 20 to 24 h in an ambient air incubator before the reading of MIC results. The strains *E. coli* ATCC 35218 and *P. aeruginosa* ATCC 27853 were used as quality control strains. The MIC was defined as the lowest concentration of antimicrobial agent that completely inhibits growth of the organism in the microdilution wells as detected by the unaided eye. The interpretation of results was determined according to the CLSI guidelines [17].

### 4.5. Determination of Antimicrobial Susceptibility Using MIC Test Strip Method

Antimicrobial susceptibilities of the 300 non-duplicated Gram-negative rods against ceftolozane-tazobactam were determined using MIC test strips (Liofilchem, Roseto degli Abruzzi, Italy) due to unavailability of the drug for evaluation using the broth microdilution method. The additional 32 ESBL-producing isolates and 101 CPE against ceftazidime-avibactam and ceftolozane-tazobactam were determined using MIC test strips (Liofilchem). Briefly, an inoculum of the bacterial strain was prepared to achieve 0.5 McFarland standard turbidity. A swab of the inoculum was streaked over Mueller Hinton agar and allowed to dry. A MIC test strip was then applied to the agar surface and incubated at 37 °C for 16–20 h. The MIC was defined as to where complete inhibition of bacterial growth was observed and interpretation of results was determined according to the Clinical and Laboratory Standards Institute (CLSI) guidelines [17].

## 5. Conclusions

Based on the susceptibility results, ceftazidime-avibactam and ceftolozane-tazobactam are good alternatives for the management of infections caused by the ESBL-producing Enterobacterales and selective strains of carbapenemase-producing Enterobacterales in Hong Kong.

## Figures and Tables

**Table 1:** Antimicrobial activity of ceftazidime-avibactam, ceftolozane-tazobactam and other comparator agents against the 300 non-duplicated isolates of aerobic Gram-negative bacteria, including 55 ESBL-producers and one CPE.

Antimicrobial Agent *^a,b^*	MIC (mg/L)			Susceptibility *^d^* (%)			
	Range	MIC_50_ *^c^*	MIC_90_ *^c^*	S	SDD	I	R
Non-MDR *E. coli* (*n* = 92)							
Ceftazidime-avibactam	≤0.016 to ≥256	0.094	0.19	100	-	-	0.0
Ceftolozane-tazobactam	≤0.016 to ≥256	0.19	0.38	98.9	-	0.0	1.1
Piperacillin-tazobactam	≤0.125 to ≥256	2	32	88.0	1.1	-	10.9
Ceftazidime	≤0.125 to ≥256	0.25	1	94.6	-	1.1	4.3
Meropenem	≤0.125 to ≥256	≤0.125	0.25	98.9	-	0.0	1.1
Cefepime	≤0.125 to ≥256	≤0.125	2	92.4	4.3	-	3.3
Colistin	≤0.125 to ≥256	0.125	0.5	-	-	97.8	2.2
ESBL-producing *E. coli* (*n* = 44)							
Ceftazidime-avibactam	≤0.016 to ≥256	0.125	0.25	100.0	-	-	0.0
Ceftolozane-tazobactam	≤0.016 to ≥256	0.25	0.5	97.7	-	0.0	2.3
Piperacillin-tazobactam	≤0.125 to ≥256	4	64	61.4	6.8	-	31.8
Ceftazidime	≤0.125 to ≥256	8	128	36.4	-	13.6	50.0
Meropenem	≤0.125 to ≥256	≤0.125	0.5	100.0	-	0.0	0.0
Cefepime	≤0.125 to ≥256	16	128	15.9	22.7	-	61.4
Colistin	≤0.125 to ≥256	0.125	0.5	-	-	97.7	2.3
Carbapenamase-producing *E. coli* (*n* = 1) *^g^*							
Ceftazidime-avibactam	≤0.016 to ≥256	-	-	0.0	-	-	100
Ceftolozane-tazobactam	≤0.016 to ≥256	-	-	0.0	-	0.0	100
Piperacillin-tazobactam	≤0.125 to ≥256	-	-	0.0	0.0	-	100
Ceftazidime	≤0.125 to ≥256	-	-	0.0	-	0.0	100
Meropenem	≤0.125 to ≥256	-	-	0.0	-	0.0	100
Cefepime	≤0.125 to ≥256	-	-	0.0	0.0	-	100
Colistin	≤0.125 to ≥256	-	-	-	-	0.0	100
Non-MDR *K. pneumoniae* (*n* = 46)							
Ceftazidime-avibactam	≤0.016 to ≥256	0.125	0.25	100.0	-	-	0.0
Ceftolozane-tazobactam	≤0.016 to ≥256	0.25	0.5	97.8	-	2.2	0.0
Piperacillin-tazobactam	≤0.125 to ≥256	0.5	64	87.0	0.0	-	13.0
Ceftazidime	≤0.125 to ≥256	1	16	84.8	-	0.0	15.2
Meropenem	≤0.125 to ≥256	0.125	0.25	100.0	-	0.0	0.0
Cefepime	≤0.125 to ≥256	0.125	0.25	97.8	2.2	-	0.0
Colistin	≤0.125 to ≥256	0.25	0.5	-	-	97.8	2.2
ESBL-producing *K. pneumoniae* (*n* = 6) *^g^*							
Ceftazidime-avibactam	≤0.016 to ≥256	-	-	100.0	-	-	0.0
Ceftolozane-tazobactam	≤0.016 to ≥256	-	-	100.0	-	0.0	0.0
Piperacillin-tazobactam	≤0.125 to ≥256	-	-	100.0	0.0	-	11.3
Ceftazidime	≤0.125 to ≥256	-	-	33.3	-	0.0	66.7
Meropenem	≤0.125 to ≥256	-	-	100.0	-	0.0	0.0
Cefepime	≤0.125 to ≥256	-	-	33.3	16.7	-	50.0
Colistin	≤0.125 to ≥256	-	-	-	-	100.0	0.0
*P. aeruginosa* (*n* = 35)							
Ceftazidime-avibactam	≤0.016 to ≥256	1.5	3.0	100.0	-	-	0.0
Ceftolozane-tazobactam	≤0.016 to ≥256	0.5	2.0	94.2	-	2.9	2.9
Piperacillin-tazobactam	≤0.125 to ≥256	16	256	62.9	-	8.6	28.5
Ceftazidime	≤0.125 to ≥256	2	64	80.0	-	0.0	20.0
Meropenem	≤0.125 to ≥256	2	64	65.7	-	8.6	25.7
Cefepime	≤0.125 to ≥256	4	16	85.7	-	5.7	8.6
Colistin	≤0.125 to ≥256	1	4	-	-	88.6	11.4
*Enterobacter* spp. (*n* = 9) *^g^*							
Ceftazidime-avibactam	≤0.016 to ≥256	-	-	100.0	-	-	0.0
Ceftolozane-tazobactam	≤0.016 to ≥256	-	-	100.0	-	0.0	0.0
Piperacillin-tazobactam	≤0.125 to ≥256	-	-	88.9	0.0	-	11.1
Ceftazidime	≤0.125 to ≥256	-	-	88.9	-	0.0	11.1
Meropenem	≤0.125 to ≥256	-	-	100.0	-	0.0	0.0
Cefepime	≤0.125 to ≥256	-	-	100.0	0.0	-	0.0
Colistin	≤0.125 to ≥256	-	-	-	-	66.7	33.3
Non-MDR *P. mirabilis, Proteus* spp. (*n* = 11)							
Ceftazidime-avibactam	≤0.016 to ≥256	0.094	0.125	100.0	-	-	0.0
Ceftolozane-tazobactam	≤0.016 to ≥256	0.75	1.5	90.9	-	0.0	9.1
Piperacillin-tazobactam	≤0.125 to ≥256	0.5	64	81.8	0.0	-	18.2
Ceftazidime	≤0.125 to ≥256	0.125	16	81.8	-	0.0	18.2
Meropenem	≤0.125 to ≥256	0.25	2	72.7	-	18.2	9.1
Cefepime	≤0.125 to ≥256	0.125	1	90.9	12.5	-	9.1
Colistin	≤0.125 to ≥256	256	≥256	-	-	9.1	90.9
ESBL-producing *P. mirabilis, Proteus* spp. (*n* = 5) *^g^*							
Ceftazidime-avibactam	≤0.016 to ≥256	-	-	100.0	-	-	0.0
Ceftolozane-tazobactam	≤0.016 to ≥256	-	-	100.0	-	0.0	0.0
Piperacillin-tazobactam	≤0.125 to ≥256	-	-	100.0	0.0	-	0.0
Ceftazidime	≤0.125 to ≥256	-	-	100.0	-	0.0	0.0
Meropenem	≤0.125 to ≥256	-	-	100.0	-	0.0	0.0
Cefepime	≤0.125 to ≥256	-	-	40.0	40.0	-	20.0
Colistin	≤0.125 to ≥256	-	-	-	-	0.0	100.0
*Citrobacter* spp. (*n* = 12)							
Ceftazidime-avibactam	≤0.016 to ≥256	0.125	0.5	100.0	-	-	0.0
Ceftolozane-tazobactam	≤0.016 to ≥256	0.25	1.5	100.0	-	0.0	0.0
Piperacillin-tazobactam	≤0.125 to ≥256	2	16	83.4	8.3	-	8.3
Ceftazidime	≤0.125 to ≥256	0.125	64	83.3	-	0.0	16.7
Meropenem	≤0.125 to ≥256	0.125	0.5	100.0	-	0.0	0.0
Cefepime	≤0.125 to ≥256	0.125	0.5	100.0	0.0	-	0.0
Colistin	≤0.125 to ≥256	0.125	1	-	-	91.7	8.3
*A. baumannii* (*n* = 13)							
Ceftazidime-avibactam *^e^*	≤0.016 to ≥256	2	4	100.0	-	-	0.0
Ceftolozane-tazobactam *^e^*	≤0.016 to ≥256	≤0.016	4	61.5	-	38.5	0.0
Piperacillin-tazobactam	≤0.125 to ≥256	0.25	256	69.2	-	0.0	30.8
Ceftazidime	≤0.125 to ≥256	2	16	84.6	-	7.7	7.7
Meropenem	≤0.125 to ≥256	0.5	64	69.2	-	0.0	30.8
Cefepime	≤0.125 to ≥256	1	256	69.2	-	7.7	23.1
Colistin	≤0.125 to ≥256	0.25	0.5	-	-	100.0	0.0
*S. maltophilia* (*n* = 14)							
Ceftazidime-avibactam *^e^*	≤0.016 to ≥256	24	≥256	28.6	-	-	71.4
Ceftolozane-tazobactam *^e^*	≤0.016 to ≥256	96	≥256	14.3	-	21.4	64.3
Piperacillin-tazobactam *^f^*	≤0.125 to ≥256	≥256	≥256	0.0	-	7.1	92.9
Ceftazidime	≤0.125 to ≥256	32	≥256	21.4	-	14.3	64.3
Meropenem *^f^*	≤0.125 to ≥256	≥256	≥256	0.0	-	0.0	100.0
Cefepime *^f^*	≤0.125 to ≥256	64	256	0.0	-	21.4	78.6
Colistin *^e^*	≤0.125 to ≥256	16	64	-	-	21.4	78.6
*M. morganii* (*n* = 7) *^g^*							
Ceftazidime-avibactam	≤0.016 to ≥256	-	-	100.0	-	-	0.0
Ceftolozane-tazobactam	≤0.016 to ≥256	-	-	100.0	-	0.0	0.0
Piperacillin-tazobactam	≤0.125 to ≥256	-	-	100.0	0.0	-	0.0
Ceftazidime	≤0.125 to ≥256	-	-	71.4	-	28.6	0.0
Meropenem	≤0.125 to ≥256	-	-	100.0	-	0.0	0.0
Cefepime	≤0.125 to ≥256	-	-	85.7	14.3	-	0.0
Colistin	≤0.125 to ≥256	-	-	-	-	14.3	85.7
*P. stuartii* (*n* = 1) *^g^*							
Ceftazidime-avibactam	≤0.016 to ≥256	-	-	100.0	-	-	0.0
Ceftolozane-tazobactam	≤0.016 to ≥256	-	-	100.0	-	0.0	0.0
Piperacillin-tazobactam	≤0.125 to ≥256	-	-	100.0	0.0	-	0.0
Ceftazidime	≤0.125 to ≥256	-	-	100.0	-	0.0	0.0
Meropenem	≤0.125 to ≥256	-	-	100.0	-	0.0	0.0
Cefepime	≤0.125 to ≥256	-	-	100.0	0.0	-	0.0
Colistin	≤0.125 to ≥256	-	-	-	-	0.0	100.0
*Serratia* spp. (*n* = 2) *^g^*							
Ceftazidime-avibactam	≤0.016 to ≥256	-	-	100.0	-	-	0.0
Ceftolozane-tazobactam	≤0.016 to ≥256	-	-	100.0	-	0.0	0.0
Piperacillin-tazobactam	≤0.125 to ≥256	-	-	100.0	0.0	-	0.0
Ceftazidime	≤0.125 to ≥256	-	-	50.0	-	0.0	50.0
Meropenem	≤0.125 to ≥256	-	-	100.0	-	0.0	0.0
Cefepime	≤0.125 to ≥256	-	-	100.0	0.0	-	0.0
Colistin	≤0.125 to ≥256	-	-	-	-	0.0	100.0
*Aeromonas* spp. (*n* = 2) *^g^*							
Ceftazidime-avibactam *^e^*	≤0.016 to ≥256	-	-	100.0	-	-	0.0
Ceftolozane-tazobactam *^e^*	≤0.016 to ≥256	-	-	50.0	-	50.0	0.0
Piperacillin-tazobactam	≤0.125 to ≥256	-	-	50.0	-	0.0	50.0
Ceftazidime	≤0.125 to ≥256	-	-	50.0	-	0.0	50.0
Meropenem	≤0.125 to ≥256	-	-	50.0	-	0.0	50.0
Cefepime	≤0.125 to ≥256	-	-	100.0	-	0.0	0.0
Colistin *^e^*	≤0.125 to ≥256	-	-	-	-	100.0	0.0

*^a^* Avibactam and tazobactam were tested at a constant concentration of 4 mg/L; breakpoints are expressed as the respective ceftazidime and ceftolozane or piperacillin component. *^b^* Table represents antimicrobial susceptibilities assessed by MIC strip test for ceftazidime-avibactam and ceftolozane-tazobactam, and broth microdilution for all other antimicrobial agents. *^c^* MIC_50_, concentration required to inhibit the growth of 50% of the isolates tested; MIC_90_, concentration required to inhibit the growth of 90% of the isolates tested. *^d^* Interpretation based on CLSI guidelines [17]. S, susceptible; SDD, susceptible dose dependent; I, intermediate; R, resistant. *^e^* Interpretation based on CLSI guidelines for Enterobacterales [17]. Dot plots of the distribution of MICs observed for these species are shown in Supplementary Figure S1. *^f^* Interpretation based on CLSI guidelines for non-Enterobacterales [17]. *^g^* No MIC_50_ or MIC_90_ value available for these species as the group had fewer than 10 isolates.

**Table 2:** Antimicrobial activity of ceftazidime-avibactam and ceftolozane-tazobactam against the additional 32 ESBL-producing and 101 CPE strains.

Antimicrobial Agent *^a^*	MIC (mg/L)			Susceptibility *^c^* (%)		
	Range	MIC_50_ *^b^*	MIC_90_ *^b^*	S	I	R
ESBL-producers (*n* = 32)						
* K. pneumoniae* (*n* = 32)						
Ceftazidime-avibactam	≤0.016 to ≥256	0.19	0.5	100.0	-	0.0
Ceftolozane-tazobactam	≤0.016 to ≥256	0.38	0.75	100.0	0.0	0.0
CPE (*n* = 101)						
* E. coli* (*n* = 50)						
Ceftazidime-avibactam	≤0.016 to ≥256	≥256	≥256	12.0	-	88.0
Ceftolozane-tazobactam	≤0.016 to ≥256	≥256	≥256	10.0	2.0	88.0
* Klebsiella* spp. (*n* = 38)						
Ceftazidime-avibactam	≤0.016 to ≥256	0.5	≥256	84.2	-	15.8
Ceftolozane-tazobactam	≤0.016 to ≥256	12	≥256	42.1	0.0	57.9
* Citrobacter* spp. (*n* = 6) *^d^*						
Ceftazidime-avibactam	≤0.016 to ≥256	-	-	50.0	-	50.0
Ceftolozane-tazobactam	≤0.016 to ≥256	-	-	50.0	0.0	50.0
* Enterobacter* spp. (*n* = 7) *^d^*						
Ceftazidime-avibactam	≤0.016 to ≥256	-	-	28.6	-	71.4
Ceftolozane-tazobactam	≤0.016 to ≥256	-	-	28.6	0.0	71.4

*^a^* Avibactam and tazobactam were tested at a constant concentration of 4 mg/L; breakpoints are expressed as the respective ceftazidime and ceftolozane. *^b^* MIC_50_, concentration required to inhibit the growth of 50% of the isolates tested; MIC_90_, concentration required to inhibit the growth of 90 of the isolates tested. *^c^* Interpretation based on CLSI guidelines [17]. S, susceptible; I, intermediate; R, resistant. *^d^* No MIC_50_ or MIC_90_ value available for these species as the group had fewer than 10 isolates.

**Table 3:** Antimicrobial activity of ceftazidime-avibactam and ceftolozane-tazobactam against the additional 101 CPE strains according to the carbapenemase gene present.

Antimicrobial Agent *^a^*	MIC (mg/L)			Susceptibility *^c^* (%)		
	Range	MIC_50_ *^b^*	MIC_90_ *^b^*	S	I	R
*NDM-positive* (*n* = 55)						
Ceftazidime-avibactam	≤0.016 to ≥256	≥256	≥256	0.0	-	100.0
Ceftolozane-tazobactam	≤0.016 to ≥256	≥256	≥256	0.0	0.0	100.0
*OXA-positive* (*n* = 34)						
Ceftazidime-avibactam	≤0.016 to ≥256	0.25	0.75	97.1	-	2.9
Ceftolozane-tazobactam	≤0.016 to ≥256	1.5	24	76.5	2.9	20.6
*NDM+OXA-positive* (*n* = 2) *^d^*						
Ceftazidime-avibactam	≤0.016 to ≥256	-	-	0.0	-	100.0
Ceftolozane-tazobactam	≤0.016 to ≥256	-	-	0.0	0.0	100.0
*KPC-positive* (*n* = 10)						
Ceftazidime-avibactam	≤0.016 to ≥256	1	1.5	100.0	-	0.0
Ceftolozane-tazobactam	≤0.016 to ≥256	48	64	0.0	0.0	100.0

*^a^* Avibactam and tazobactam were tested at a constant concentration of 4 mg/L; breakpoints are expressed as the respective ceftazidime and ceftolozane. *^b^* MIC_50_, concentration required to inhibit the growth of 50% of the isolates tested; MIC_90_, concentration required to inhibit the growth of 90% of the isolates tested. *^c^* Interpretation based on CLSI guidelines [17]. S, susceptible; I, intermediate; R, resistant. *^d^* No MIC_50_ or MIC_90_ value available as the group had fewer than 10 isolates.

## Data Availability

Data are contained within the article and Supplementary Materials.

## Supplementary Materials

The following supporting information can be downloaded at: https://www.mdpi.com/article/10.3390/antibiotics13090802/s1, Figure S1: Dot plots of distribution of MICs observed in *A. baumannii* (*n* = 13), *Aeromonas* spp. (*n* = 2), and *S. maltophilia* (*n* = 14) for (A) ceftazidime-avibactam and (B) ceftolozane-tazobactam, both evaluated using MIC test strips, and (C) colistin, evaluated using broth microdilution. Avibactam and tazobactam were tested at a constant concentration of 4 mg/L, breakpoints are expressed as the respective ceftazidime and ceftolozane component. Each dot represents one tested isolate; Table S1: Patient isolation sites of the 300 Gram-negative bacterial strains, including 55 ESBL-producers, collected in this study; Table S2: Patient isolation sites of the additional 32 ESBL-producing and 101 CPE strains that were collected for further evaluation of antimicrobial susceptibilities to ceftazidime-avibactam and ceftolozane-tazobactam. Table S3: Comparison of antimicrobial activity of ceftazidime-avibactam assessed using MIC test strips and broth microdilution method.
