# Supplementary material for: Antibiotic Susceptibility of Aerobic and Facultative Anaerobic Gram-Negative Rods in Hong Kong and Implications on Usefulness of Ceftazidime-Avibactam and Ceftolozane-Tazobactam
Source: Antibiotics (Basel). 2024 Aug 24;13(9):802. doi: 10.3390/antibiotics13090802 (PMC11428689; doi:10.3390/antibiotics13090802)
Supplement: Supplementary file 1 [file antibiotics-13-00802-s001.zip › Manuscript_Pfizer_Supplementary Tables_20240804.pdf]

Supplementary Table S1. Patient isolation sites of the 300 Gram-negative bacterial strains, including 55 ESBL-producers, collected in this study.

Organism	**Number of strains from isolation site**			
	Urine	*Respiratory samples*	*Miscellaneous samples*	*Blood*
Gram-negative isolates (*n* = 300)				
E. coli (*n* = 137)	41	12	46	38
K. pneumoniae (*n* = 52)	14	15	16	7
P. aeruginosa (*n* = 35)	0	35	0	0
Enterobacter spp. (*n* = 9)	0	6	2	1
P. mirabilis (*n* = 14)	0	4	5	5
Proteus spp. (*n* = 2)	0	0	1	1
Citrobacter spp. (*n* = 12)	3	1	6	2
A. baumannii (*n* = 13)	0	12	0	1
S. maltophilia (*n* = 14)	0	11	2	1
M. morganii (*n* = 7)	2	1	3	1
P. stuartii (*n* = 1)	0	0	1	0
Serratia spp. (*n* = 2)	0	2	0	0
Aeromonas spp. (*n* = 2)	0	0	2	0
ESBL-producers (*n* = 55)				
E. coli (*n* = 44)	18	5	10	11
K. pneumoniae (*n* = 6)	1	2	3	0
P. mirabilis (*n* = 4)	0	1	2	1
Proteus spp. (*n* = 1)	0	0	0	1

Supplementary Table S2. Patient isolation sites of the additional 32 ESBL-producing and 101 CPE strains that were collected for further evaluation of antimicrobial susceptibilities to ceftazidime-avibactam and ceftolozane-tazobactam.

**Organism**	**Number of strains from isolation site**				
	Urine	*Respiratory samples*	*Miscellaneous samples*	*Stool*	*Blood*
ESBL-producers (*n* = 32)					
K. pneumoniae (*n* = 32)	32	0	0	0	0
CPE strains (*n* = 101)					
E. coli (*n* = 50)	31	4	11	1	3
Klebsiella spp. (*n* = 38)	22	6	4	1	5
Citrobacter spp. (*n* = 6)	5	0	0	0	1
Enterobacter spp. (*n* = 7)	4	2	0	0	1

Supplementary Table S3. Comparison of antimicrobial activity of ceftazidime-avibactam assessed using MIC test strips and broth microdilution method

**Bacterial isolate**	**MIC test strips**[a]			**Broth microdilution**[a]		
	**MIC_{50}**[b]	**MIC_{90}**[b]	**% susceptible**[c]	**MIC_{50}**[b]	**MIC_{90}**[b]	**% susceptible**[c]
Non-MDR *E. coli* (*n* = 92)	0.094	0.19	100.0	0.125	0.5	100.0
ESBL-producing E. coli (*n* = 44)	0.125	0.25	100.0	0.25	1	100.0
Carbapenamase-producing E. coli (*n* = 1)[d]	-	-	0.0	-	-	0.0
Non-MDR *K. pneumoniae* (*n* = 46)	0.125	0.25	100.0	0.125	0.5	100.0
ESBL-producing *K. pneumoniae* (*n* = 6)[d]	-	-	100.0	-	-	100.0
P. aeruginosa (*n* = 35)	1.5	3.0	100.0	2	4	100.0
Enterobacter spp. (*n* = 9)[d]	-	-	100.0	-	-	100.0
Non-MDR *P. mirabilis, Proteus* spp. (*n* = 11)	0.094	0.125	100.0	0.125	0.125	100.0
ESBL-producing *P. mirabilis, Proteus* spp. (*n* = 5)[d]	-	-	100.0	-	-	100.0
Citrobacter spp. (*n* = 12)	0.125	0.5	100.0	0.125	0.5	100.0
A. baumannii (*n* = 13)	2	4	100.0	4	8	100.0
S. maltophilia (*n* = 14)	24	≥256	28.6	16	256	35.7
M. morganii (*n* = 7)[d]	-	-	100.0	-	-	100.0
P. stuartii (*n* = 1)[d]	-	-	100.0	-	-	100.0
Serratia spp. (*n* = 2)[d]	-	-	100.0	-	-	100.0
Aeromonas spp. (*n* = 2)[d]	-	-	100.0	-	-	100.0

[a]Avibactam was tested at a constant concentration of 4 mg/L, breakpoints are expressed as the ceftazidime component.

[b]MIC_{50}, concentration required to inhibit the growth of 50% of the isolates tested; MIC_{90}, concentration required to inhibit the growth of 90% of the isolates tested.

[c]Interpretation based on CLSI guidelines [13]. S, susceptible; I, intermediate; R, resistant.

[d]No MIC_{50} and MIC_{90} values available for these species as the group had less than 10 isolates.
