# Supplementary material for: Antibiotic Susceptibility of Aerobic and Facultative Anaerobic Gram-Negative Rods in Hong Kong and Implications on Usefulness of Ceftazidime-Avibactam and Ceftolozane-Tazobactam
Source: Antibiotics (Basel). 2024 Aug 24;13(9):802. doi: 10.3390/antibiotics13090802 (PMC11428689; doi:10.3390/antibiotics13090802)
Supplement: Supplementary file 1 [file antibiotics-13-00802-s001.zip › Supplementary Figure S1_20240809.pdf]

Supplementary Figure S1. Dot plots of distribution of MICs observed for selected non-Enterobacterales, including *A. baumannii (n* = 13*), Aeromonas* spp. *(n* = 2*),* and *S. maltophilia* (n = 14), where CLSI guidelines for Enterobacterales was used to interpret MIC results for **(A)** ceftazidime-avibactam and **(B)** ceftolozane-tazobactam, both evaluated using MIC test strips, and **(C)** colistin, evaluated using broth microdilution. Enterobacterales breakpoints for colistin were used for *Aeromonas* spp. and *S. maltophilia* only. Avibactam and tazobactam were tested at a constant concentration of 4 mg/L, breakpoints are expressed as the respective ceftazidime and ceftolozane component. Each dot represents one tested isolate.

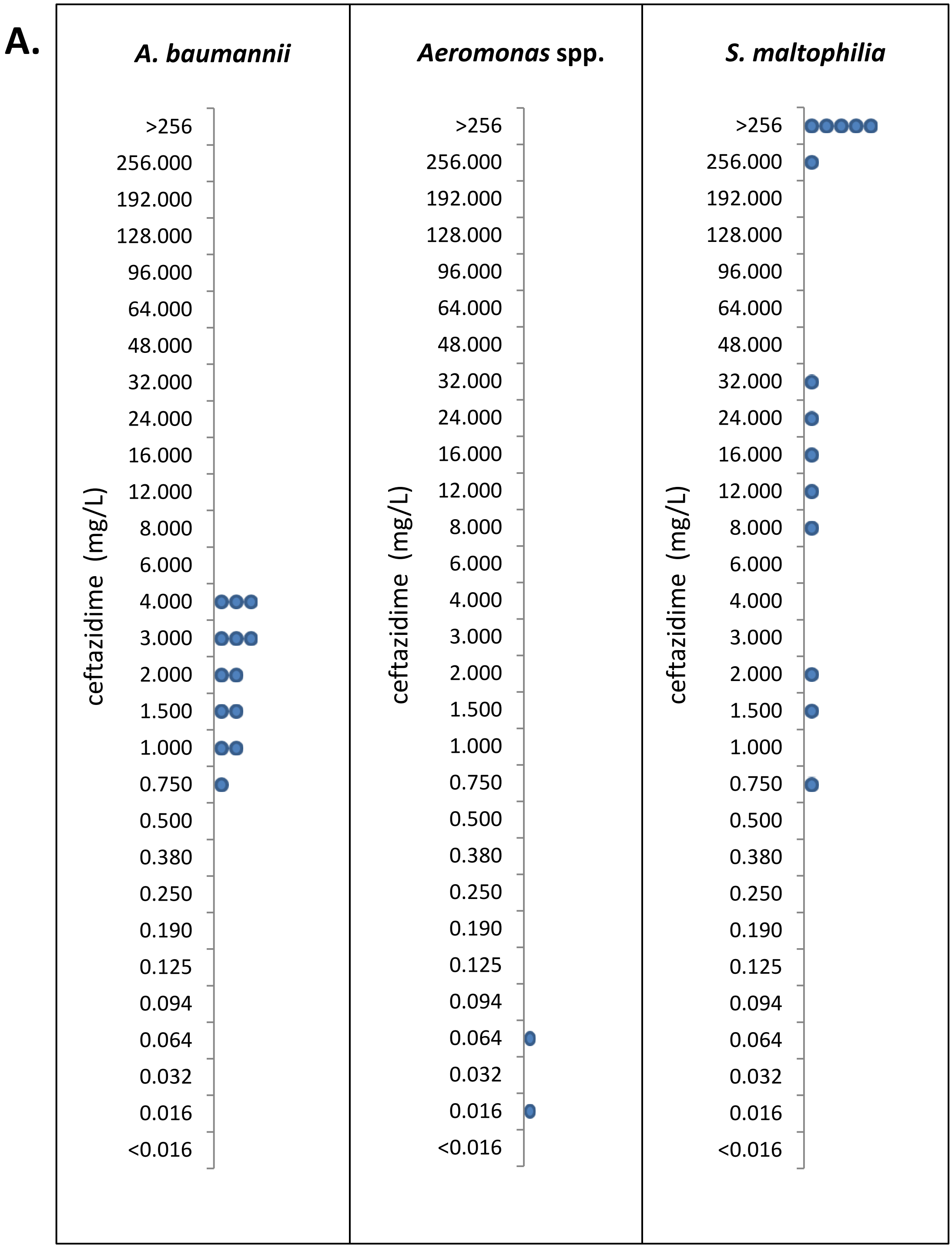

B.

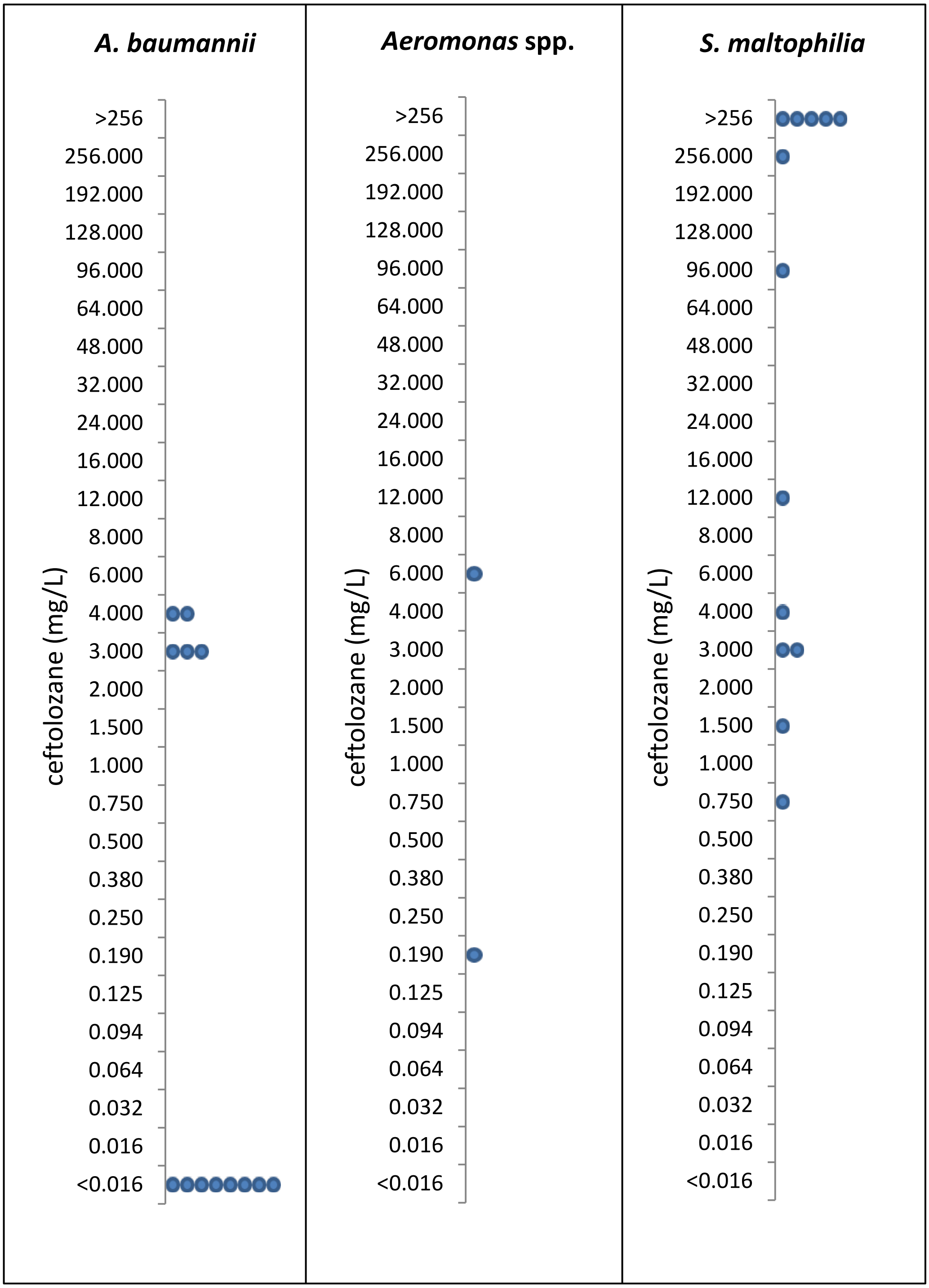
A. baumannii
Aeromonas spp.
S. maltophilia
ceftolozane (mg/L)
>256
256.000
192.000
128.000
96.000
64.000
48.000
32.000
24.000
16.000
12.000
8.000
6.000
4.000
3.000
2.000
1.500
1.000
0.750
0.500
0.380
0.250
0.190
0.125
0.094
0.064
0.032
0.016
<0.016

C.

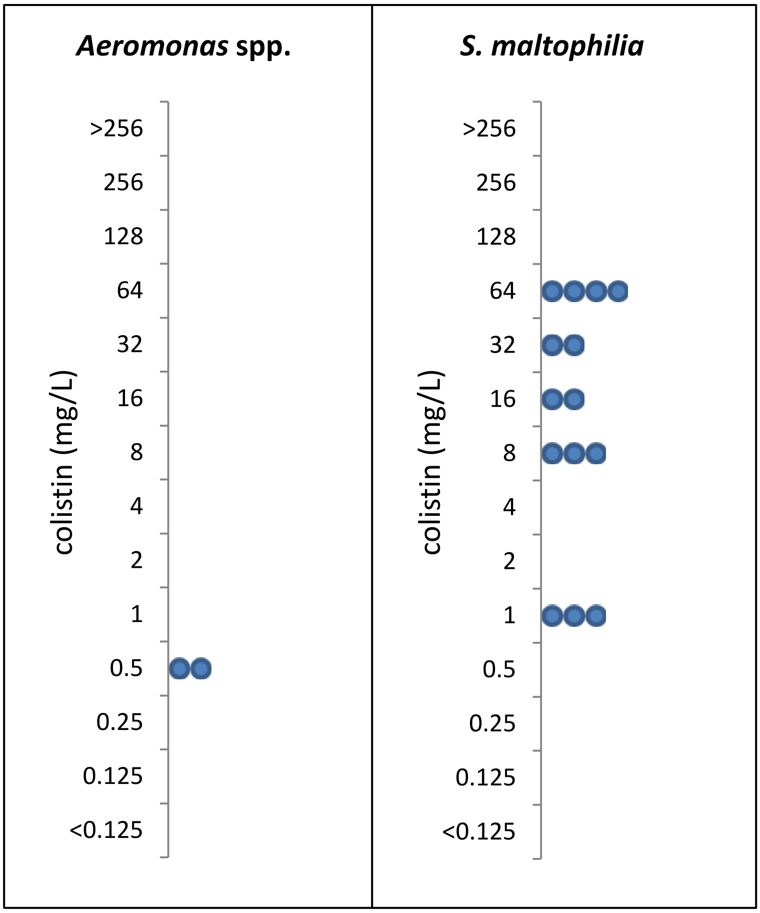

Aeromonas spp.
S. maltophilia
colistin (mg/L)
>256
256
128
64
32
16
8
4
2
1
0.5
0.25
0.125
<0.125
